# Stromal–epithelial cell interactions and alteration of branching morphogenesis in macromastic mammary glands

**DOI:** 10.1111/jcmm.12275

**Published:** 2014-04-10

**Authors:** Aimei Zhong, Guohua Wang, Jie Yang, Qijun Xu, Quan Yuan, Yanqing Yang, Yun Xia, Ke Guo, Raymund E Horch, Jiaming Sun

**Affiliations:** a Department of Plastic Surgery, Union Hospital, Tongji Medical College, Huazhong University of Science and TechnologyWuhan, Hubei, China; b Department of Cardiovascular Surgery, Union Hospital, Tongji Medical College, Huazhong University of Science and TechnologyWuhan, Hubei, China; c Department of Plastic and Hand Surgery and Laboratory for Tissue Engineering and Regenerative Medicine, Friedrich Alexander University Erlangen-NuernbergErlangen, Germany

**Keywords:** macromastia, epithelial cells, stromal cells, hepatocyte growth factor, proliferation, branching morphogenesis

## Abstract

True macromastia is a rare but disabling condition characterized by massive breast growth. The aetiology and pathogenic mechanisms for this disorder remain largely unexplored because of the lack of *in vivo* or *in vitro* models. Previous studies suggested that regulation of epithelial cell growth and development by oestrogen was dependent on paracrine growth factors from the stroma. In this study, a co-culture model containing epithelial and stromal cells was used to investigate the interactions of these cells in macromastia. Epithelial cell proliferation and branching morphogenesis were measured to assess the effect of macromastic stromal cells on epithelial cells. We analysed the cytokines secreted by stromal cells and identified molecules that were critical for effects on epithelial cells. Our results indicated a significant increase in cell proliferation and branching morphogenesis of macromastic and non-macromastic epithelial cells when co-cultured with macromastic stromal cells or in conditioned medium from macromastic stromal cells. Hepatocyte growth factor (HGF) is a key factor in epithelial–stromal interactions of macromastia-derived cell cultures. Blockade of HGF with neutralizing antibodies dramatically attenuated epithelial cell proliferation in conditioned medium from macromastic stromal cells. The epithelial–stromal cell co-culture model demonstrated reliability for studying interactions of mammary stromal and epithelial cells in macromastia. In this model, HGF secreted by macromastic stromal cells was found to play an important role in modifying the behaviour of co-cultured epithelial cells. This model allows further studies to investigate basic cellular and molecular mechanisms in tissue from patients with true breast hypertrophy.

## Introduction

Hypertrophy of the breast is a relatively rare condition leading to macromastia. It is defined as excessive hyperplasia of the mammary glands and surrounding fat tissue, with an increase in breast volume and weight (≥1500 g per breast) [1–3]. Rapid growth of one or both breasts in a woman leads to significant physical and psychological difficulties that affect appearance and can also cause terrible suffering. This disease has been suspected to be associated with increased levels of oestrogen, progesterone or related enzymes in local tissue [1,3,4], but the mechanism of breast hypertrophy remains to be elucidated. Development of the mammary ducts is caused by extensive proliferation of epithelial cells, which ultimately form dendritic breast tissue in the fat pad [5]. The growth and development of mammary epithelia are driven by breast stroma in an oestrogen-dependent manner, which has been confirmed by joint tissue trials [6–8]. This oestrogen-dependent process is associated with the paracrine effects of topical growth factors [9,10]. Consequently, disruption of this interaction may affect the development and progression of the breasts.

To investigate the underlying mechanism of macromastia, we used a three-dimensional (3D) primary co-culture system in which the cells were in an environment closely resembling that *in vivo*. Epithelial cells were embedded in a matrix and co-cultured with stromal cells from normal or pathological conditions to study the role of normal and macromastic stromal cells in the growth and morphogenesis of epithelial cells.

## Materials and methods

### Materials

Collagenase types I (catalog no. C0130) and IV (catalog no. C5138), foetal bovine serum (FBS, catalog no. F0926), hydrocortisone (catalog no. H0888), insulin (catalog no. I1882), epidermal growth factor (EGF, catalog no. E9644), bovine serum albumin (BSA, catalog no. A8022; fraction V), 17-estradiol (catalog no. E2758), anti-hepatocyte growth factor (HGF, catalog no. H0652), anti-insulin-like growth factor-1 (IGF-1, catalog no. I8773), anti-EGF (catalog no. E2520), penicillin-streptomycin (catalog no. P0781) were purchased from Sigma-Aldrich. (St. Louis, MO, USA). Percoll used in gradient centrifugation was purchased from Pharmacia Fine Chemicals (catalog no. 17-0891-01, Uppsala, Sweden). DMEM/F12 was purchased from Invitrogen (catalog no. 11330; Carlsbad, CA, USA). Mouse anti-cytokeratin 18 (CK 18, catalog no. ab82254) and rabbit anti-vimentin antibodies (catalog no. ab137321), were purchased from Abcam (Cambridge, UK). Horseradish peroxidase (HRP)-labelled (goat anti-rabbit IgG, goat antimouse IgG, donkey anti-goat IgG) and FITC-conjugated (donkey antimouse IgG, catalog no. sc-2099) antibodies were purchased from Santa Cruz Biotechnology Inc. (Santa Cruz, CA, USA). Cy3-conjugated goat anti-rabbit IgG was from Jackson ImmunoReasearch (catalog no. 111-167-003; West Grove, PA, USA). Matrigel (Growth Factor Reduced; catalog no. 356231) and mouse anti-Ki67 antibody (catalog no. 550609) were purchased from BD Biosciences (San Jose, CA, USA). Non-essential amino acids (catalog no. 11140-050) and L-glutamine (catalog no. 25030-081) were purchased from Gibco (Grand Island, NY, USA). Tritiated [^3^H] thymidine was from ICN Pharmaceuticals (catalog no. 11320; Irvine, CA, USA). SuperSignal West Pico Chemiluminescent Substrate was purchased from Thermo Scientific (catalog no. 34077; Rockford, IL, USA).

### Mammary cell isolation

Breast tissues obtained from Union Hospital (Wuhan, China) were excised from macromastia patients who underwent mammaplasty or mastectomy, and patients with early-stage breast cancer who underwent mastectomy. The patient information including age, excised breast weight and BMI has been summarized in Table 1. The excised tissues from cancer patients were far from the lesions and excluded carcinogenesis. All tissue procurement was conducted following standard procedures reviewed and approved by the Research Ethical Committee of Huazhong University of Science and Technology. Macromastic tissue was analysed by the histology/pathology laboratory at the Union Hospital and diagnosed as normal or hyperplastic. Mammary gland tissue appeared as white strands embedded in the yellow fat matrix. The white areas were dissected out and cut into 1 mm pieces under sterile conditions and subjected to digestion in collagenase (0.125% type I and 0.125% type IV collagenases) for 5 hrs at 37°C [11,12]. Stromal and epithelial cells were separated by Percoll gradient centrifugation. Stromal cells from the top layer were plated in DMEM/F12 containing 10% FBS, 100 μg/ml penicillin and 50 μg/ml streptomycin. Epithelial cells from the interface layer were plated in DMEM/F12 containing 0.5 μg/ml hydrocortisone, 5 μg/ml insulin, 10 ng/ml EGF, 0.1 g/ml BSA, 20 nm 17-estradiol, 100 μg/ml penicillin and 50 μg/ml streptomycin.

**Table 1:** Samples information

Samples	Diagnosis	Age (years)	Weight of excised breast(s)* (kg)	Body mass index (BMI, kg/m^2^)	Management
Macromastia epithelial/stromal cells	Macromastia	36	3.75	28.5	Bilateral reduction mammoplasty
	Macromastia	32	3.91	27.1	Bilateral reduction mammoplasty
	Macromastia	35	4.13	29.3	Mastectomy
	Macromastia	26	3.78	30.2	Bilateral reduction mammoplasty
Non-macromastia epithelial/stromal cells	Mammary ductal carcinoma *in situ*	26	1.10	22.1	Mastectomy
	Mammary ductal carcinoma *in situ*	30	1.45	24.4	Mastectomy
	Mammary ductal carcinoma *in situ*	32	1.36	23.7	Mastectomy
	Mammary ductal carcinoma *in situ*	29	1.22	22.6	Mastectomy

* Weight of excised breasts for macromastia was from both breasts, while weight of excised breast for non-macromastia group was from one breast that was treated by mastectomy.

**Table 2:** Morphology and proliferation of mammary epithelial cells

Co-culture			

Epithelial cells	Stromal cells	Organoid size (pixels/organoid)	[^3^H]-TdR Inc. (cpm/cell)
M-epithelial	NM-Stromal	7490 ± 2159	0.148 ± 0.006
M-epithelial	M-Stromal	17062 ± 2370^a^	0.252 ± 0.019^b^
NM-epithelial	NM-Stromal	6356 ± 2038	0.135 ± 0.015
NM-epithelial	M-Stromal	10945 ± 1950^a^	0.188 ± 0.012^b^

a *P* < 0.01, organoid size for M-epithelial or NM-epithelial was increased significantly (2.3- and 1.7-fold, respectively) when co-cultured with M-stromal compared with NM-stromal;

b *P* < 0.05, [^3^H]-TdR Inc. of M-epithelial or NM-epithelial decreased significantly (41% and 28%, respectively) when co-cultured with conditional medium (CM) from NM-stromal, compared with CM from M-stromal. M, Macromastia; NM, non-macromastia; [^3^H]-TdR Inc., [^3^H] thymidine incorporation.

### Immunohistochemistry and immunocytochemistry

Mammary tissue specimens were fixed with formalin and embedded in paraffin. Cultured cells were fixed *in situ* with 4% (w/v) paraformaldehyde. Histological sections and fixed cells were immunostained using anti-CK18 (1:1000) and/or anti-vimentin (1:2000) antibodies. HRP-conjugated goat anti-rabbit IgG (1:200) and goat antimouse IgG (1:200) were used as secondary antibodies. Haematoxylin was used for counterstaining. For immunofluorescence, cells were incubated with Cy3-conjugated goat anti-rabbit IgG (1:100) and FITC-conjugated donkey antimouse IgG (1:100). Nuclei were counterstained with 4′,6-diamidino-2-phenylindole (DAPI). Signals were detected by fluorescence microscopy. Primary antibodies were omitted for negative controls.

### Preparation of conditioned medium (CM)

Stromal cells were seeded in 6-well plates and cultured in DMEM/F12 supplemented with 10% FBS. Sub-confluent cultures were washed twice with PBS and incubated in basal medium (phenol red-free DMEM/F12 containing 0.1 mM non-essential amino acids, 2 mM L-glutamine, 100 ng/ml insulin, 1 mg/ml BSA, 100 μg/ml penicillin and 50 μg/ml streptomycin) for 48 hrs. Conditioned medium was collected, centrifuged at 1500 × g for 10 min. at 4°C, passed through a 0.22 μm filter and stored at 4°C for up to 1 month. The volume of CM used in each experiment was normalized according to the number of cells present. In some experiments, CM was incubated with neutralizing antibodies for 2 hrs at 37°C before applying to cell cultures.

### 3D co-culture

Second-passage stromal cells (5 × 10^3^ cells/well, 96-well plates) from macromastic or non-macromastic breast tissues were plated in DMEM/F12 containing 10% FBS. The medium was removed after 24 hrs in culture, and the cells were washed twice with PBS. The cells were then covered with Matrigel (1:1 dilution, 25 μl/well). After 45 min. at 37°C, second-passage epithelial cells (1 × 10^4^ cells/well) from macromastic or non-macromastic tissues were suspended in Matrigel, then plated on top of the stromal layer, incubated for 45 min. at 37°C and then covered with basal medium. Procedures involving Matrigel were performed on ice according to the manufacturer's instructions. Cultures were maintained at 37°C with 5% CO_2_ for up to 10 days with the medium changed every 2 days.

### Branching morphogenesis

Organoid morphology in Matrigel was visualized *in situ* with the aid of an inverted phase-contrast microscope and Spot camera. For each experimental condition, number of organoids was counted and 15 organoids were randomly chosen from culture wells. Images of organoids were captured and the area per organoid was determined by NIH ImageJ software.

### Analysis of epithelial cell proliferation

Isolated epithelial cells were seeded in triplicate at 8000 cells/well in 96-well plates and cultured with CM from macromastic or non-macromastic stromal cells. After 24 hrs of culture, DNA synthesis was determined using [^3^H] thymidine incorporation assays. The cells were incubated with [^3^H] thymidine at a final concentration of 2.5 μCi/ml [13] for 6 hrs at 37°C and then washed twice with Hank's balanced salt solution. Cells were then fixed with 5% trichloroacetic acid (TCA) for 20 min. at 4°C and then rinsed three times with 5% TCA. After air drying, cells were dissolved in 0.2 M NaOH for 30 min. and then neutralized with HCl. Radioactivity was detected by liquid scintillation counting. Thymidine incorporation was standardized according to total cell counts. For Ki67 staining, epithelial cells were seeded on coverslips and cultured with CM from macromastic or non-macromastic stromal cells. After 24 hrs of culture, cells were fixed and immunostained using mouse anti-Ki67 (1:200) antibody, then incubated with FITC-conjugated donkey antimouse IgG (1:100). Nuclei were counterstained with DAPI.

### Western blot analysis of IGF-1, EGF and HGF in CM

To evaluate the growth factors in CM of stromal cells from macromastia and non-macromastia tissues, CM from each stromal cell population was concentrated fourfold using a Microcon centrifugal filter device (3-kD cut-off; Millipore, Bedford, MA, USA). Proteins in the concentrated CM were subjected to electrophoresis on sodium dodecyl sulphate polyacrylamide gel and then transferred to a nitrocellulose membrane. After blocking, membranes were probed overnight with goat anti-human HGF (1:2000), goat anti-human IGF-1 (1:2000) or mouse anti-human EGF (1:2000) antibodies at 4°C. After washing, the appropriate HRP-labelled secondary antibody (1:5000) was added to the membranes. Immune complexes were developed using a Super Signal West Pico Chemiluminescence Substrate and then exposed to film to visualize the protein bands.

### Statistics

Three independent experiments were assessed at least and data were expressed as the mean ± the standard error of the mean (SEM) or standard deviation (SD), and evaluated for significance using the Student's *t*-test or anova.

## Results

### Isolation of epithelial and stromal cells from fresh human mammary tissue

**Fig. 1:**
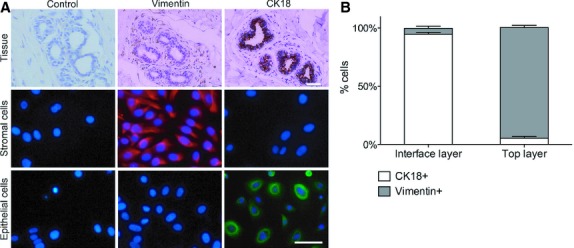
Immunocytochemical analysis of mammary epithelial and stromal cell markers. (**A**) Tissue sections (100×) and primary cell cultures of stromal and epithelial cells (200×) were fixed and stained for vimentin and cytokeratin 18 (CK18). Negative controls were provided without a primary antibody. The populations divided by centrifugation in discontinuous Percoll gradients were characterized for epithelial (CK18) and stromal cellular makers (vimentin); scale bar: 50 μm. (**B**) Quantification of staining was shown as the percentage that equal to the number of CK18/Vimentin positive or negative cells divided by the number of cell nuclei (*n* = 5, values represent mean ± SEM).

**Fig. 2:**
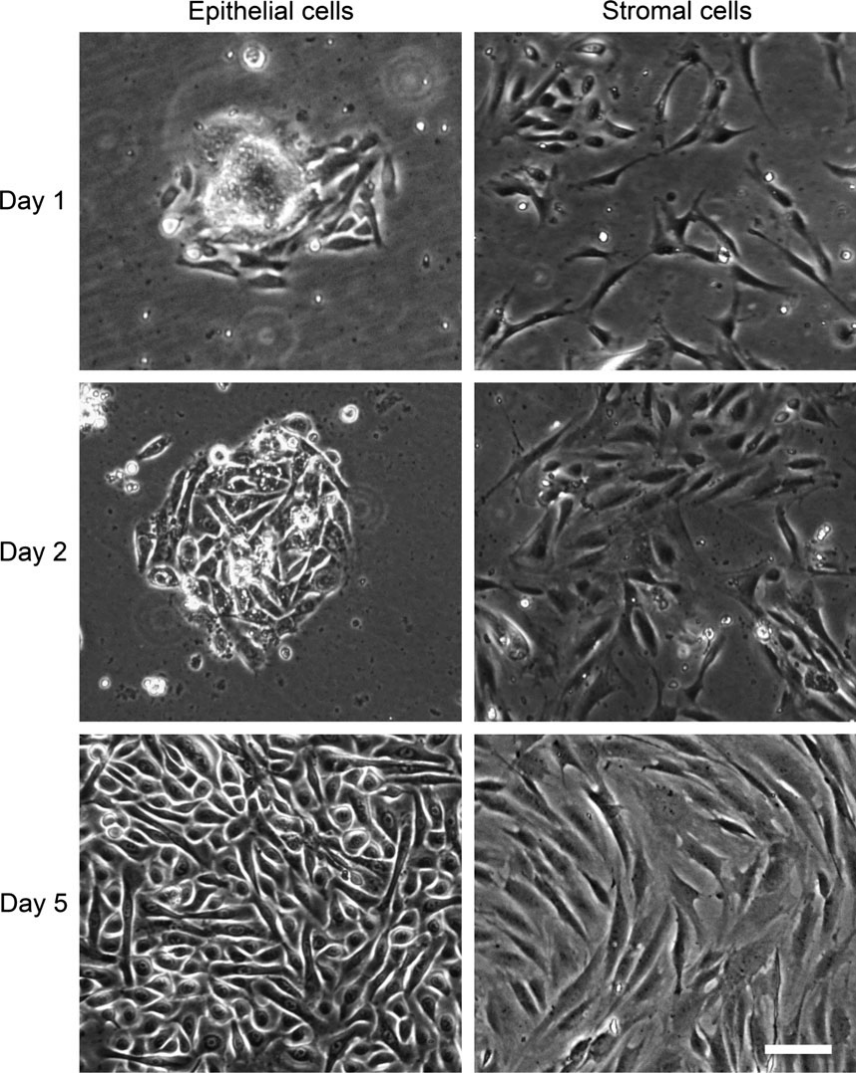
Phase-contrast photomicrographs of cultured mammary epithelial and stromal cells separated by Percoll gradients (100×). Mammary epithelial cells were mostly short spindle or cubical-like cobblestone and closely aligned with each other, linking into pieces at days 1, 2 and 5 after seeding. For comparison, the growth of stromal cells mostly appeared as strips, or swirling and elongated, at days 1, 2 and 5 after seeding; scale bar: 50 μm.

Two distinct cell populations were isolated from digested mammary glands after centrifugation in discontinuous Percoll gradients. These populations were characterized for epithelial and stromal cell markers by immunofluorescence. The interface layer was enriched for mammary epithelial cells as identified by strong immunostaining for CK18 [14]. The number of CK18 negative or positive cells was counted from the staining. Confounding of stromal cells in epithelial cultures was less than 5% as shown the number of negative staining cells for CK18 divided by the number of cells nuclei (DAPI). Cells in the top layer were cultured in serum-containing medium and found to be rich in vimentin positive cells (95 ± 2%, *n* = 5), indicating the presence of stromal cells in this population (Fig. 1). Isolated epithelial and stromal cells were expanded in culture. Purified mammary epithelial cells were mostly short spindle or cubical with a cobblestone appearance. Compared with the epithelial cells, the stromal cells mostly appeared as strips or swirls, or arranged radially (Fig. 2).

### Macromastic stromal cells improve the morphology of mammary epithelial cells

**Fig. 3:**
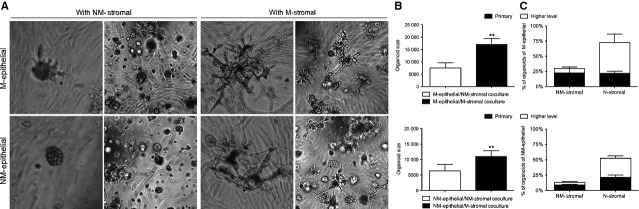
Effect of stromal cells from different origins (macromastia and non-macromastia tissues) on the morphology of mammary epithelial cells. Epithelial cells from macromastia (M-epithelial) or non-macromastia (NM-epithelial) tissues were embedded in Matrigel, and plated on top of macromastia stromal cells (M-stromal) or non-macromastia stromal cells (NM-stromal). (**A**) Phase-contrast photomicrographs of epithelial organoids in Matrigel *in situ*. Low (40×, 2nd and 4th column) and high (100×, 1st and 3rd column) magnification of the organoids were shown. (**B**) Average organoid size of epithelial co-cultured with M-stromal or NM-stromal. Organoid size of M-epithelial or NM-epithelial was significantly increased when co-cultured with M-stromal compared with NM-stromal. Values represent mean ± SD, *n* = 15. **P < 0.01. (**C**) Percentage of organoids that undergo primary (1st) or higher level (2nd and more) branching by co-cultured with M-stromal or NM-stromal. Values represent mean ± SEM, *n* = 5.

To investigate the effects of stromal cells from different origins (macromastia and non-macromastia tissues) on the morphology of mammary epithelial cells, we established 3D co-cultures of human mammary epithelial cells from macromastia or non-macromastia tissues with mammary stromal cells from macromastia or non-macromastia tissues. Macromastic epithelial cells cultured with non-macromastic stromal cells grew as round organoids for up to 10 days (7490 ± 2159 pixels/organoid, *n* = 15). In contrast, when co-cultured with macromastic stromal cells, the macromastic epithelial organoids produced several projections from day 3, which is characteristic of tubular/ductal morphology. When co-cultured for 10 days, these projections increased in number and became longer, accompanied by a 2.3-fold increase in organoid size (17062 ± 2370 pixels/organoid, *n* = 15) compared with that in co-cultures with non-macromastic stromal cells (*P* < 0.01; Fig. 3A and B). In addition to organoid size, the number of branching organoids was also increased when co-cultured with macromastia stroma (Fig. 3A and C). Non-macromastic epithelial cells had a similar round appearance when cultured with non-macromastic stromal cells for up to 10 days (6356 ± 2038 pixels/organoid, *n* = 15). In contrast, when they were co-cultured with macromastic stromal cells, the organoids displayed more projections (10,945 ± 1950 pixels/organoid, *n* = 15) and increased 1.7-fold in size (*P* < 0.01; Fig. 3B). Consistent with these results, the percentage of branching organoids was also increased by macromastic stromal cells (Fig. 3A and C).However, these projections and the number of organoids were not as apparent as those in macromastic epithelial cells co-cultured with macromastic stromal cells. Because epithelial cells were separated from stromal cells by Matrigel in the model, these morphogenic effects were believed to be essentially dependent on soluble factors that acted in a paracrine fashion. This hypothesis was tested using CM obtained from macromastic stromal cells to culture the epithelial cells.

### Effect of mammary stromal cells on proliferation of mammary epithelial cells

**Fig. 4:**
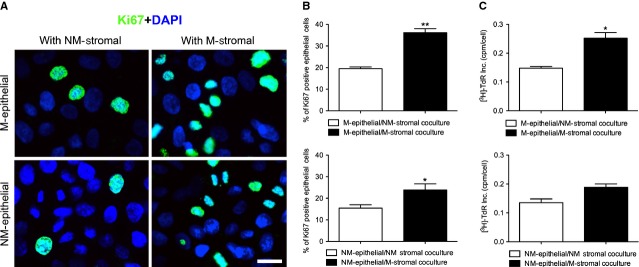
Effect of M-stromal or NM-stromal on the proliferation of mammary epithelial cells. (**A**) Ki67 staining (green) revealed proliferation of epithelial cells co-cultured with M-stromal or NM-stromal. Nuclei were stained with DAPI (blue); scale bar: 50 μm. (**B**) Quantification of Ki67 proliferation was shown as the ratio of Ki67 positive nuclei and all nuclei present in a visual field. Values represent mean ± SEM, *n* = 5. *P < 0.05; **P < 0.01. (**C**) DNA synthesis of co-cultured epithelial cells was determined by [^3^H] thymidine incorporation after 24 hrs of co-culture. Values represent mean ± SEM, *n* = 3. *P < 0.05.

To investigate the contribution of the stroma to epithelial cell growth, we analysed the growth characteristics of isolated epithelial cells cultured in CM of stromal cells from non-macromastia and macromastia tissues. There were apparent differences between primary cultures of epithelial cells in response to stromal cells obtained from macromastia and non-macromastia tissues. When cultured in CM from non-macromastia stromal cells, the proliferation of macromastic epithelial cells decreased by 41%, as measured by [^3^H]-thymidine incorporation, compared with that in CM from macromastia stromal cells (*P* < 0.05). There was a similar response in non-macromastia epithelial cells that showed a comparable reduction in proliferation when cultured in CM from non-macromastic stromal cells (28%, *P* < 0.05) compared with that in CM from macromastic stromal cells, which was consistent with the increase in organoid size by macromastic stromal cells (Fig. 4C). Ki67 staining for epithelial cells cultured with CM from different stromal cells further supported that macromastic stroma increased the proliferation of epithelial cells (Fig. 4A and B).

### Characterization of stromal-derived growth factors

**Fig. 5:**
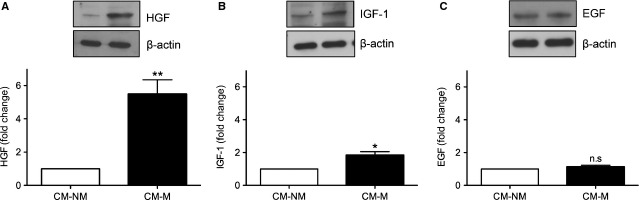
Growth factors produced by macromastia stromal cells improved the proliferation of epithelial cells. Immunoblot of insulin-like growth factor-1 (IGF-1), epidermal growth factor (EGF) and hepatocyte growth factor (HGF) in conditioned medium (CM) from macromastic (CM-M) and non-macromastic (CM-NM) stromal cells. Densitometry was conducted to determine the relative expression, and then normalized to β-actin expression in stromal cells from each well. Quantification of densitometry was shown. (**A**) Increased HGF in CM-M (**P < 0.01); (**B**) increased IGF-1 in CM-M (*P < 0.05); (**C**) EGF: no significant difference (n.s). Values represent mean ± SEM, *n* = 3.

**Fig. 6:**
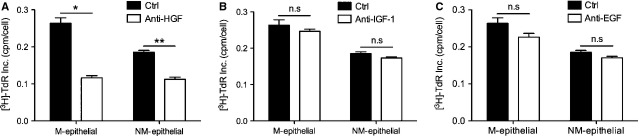
Effect of neutralizing antibodies on proliferation. Macromastia epithelial cells (M-epithelial) and non-macromastia (NM-epithelial) cells were seeded and cultured with CM-M or CM-NM pre-incubated with hepatocyte growth factor (HGF; anti-HGF), insulin-like growth factor-1 (IGF-1; anti-IGF-1) or epidermal growth factor (EGF; anti-EGF) neutralizing antibodies. (**A**) In the presence of anti-HGF, the proliferative effects of CM-M on M-epithelial (*P < 0.05) or NM-epithelial (**P < 0.01) were significantly reduced. In contrast to the pronounced proliferative reduction observed with anti-HGF, no significant changes were observed when (**B**) anti-IGF-1 or (**C**) anti-EGF were present. Values represent mean ± SEM, *n* = 3.

Several stromal cell-derived growth factors, such as HGF, IGF-1 and EGF, are important for the growth and development of epithelial cells in the mammary gland [15–17]. To determine whether these growth factors were involved in the enhanced growth and morphology induced by macromastic stromal cells, we conducted Western blot analysis to examine the expression of HGF, IGF-1 and EGF in CM of macromastic and non-macromastic stromal cells. We found that macromastic stromal cells secreted significantly more HGF and IGF-1 than non-macromastic stromal cells (Fig. 5A and B), and the expression of EGF was similar (Fig. 5C). Conditioned medium from stromal cells was pre-treated with neutralizing antibodies against HGF, IGF-1 or EGF, and then the proliferative activity of epithelial cells was assayed. The proliferative activity of CM from macromastic stromal cells was completely attenuated by the anti-HGF antibody (Fig. 6A), whereas anti-EGF and anti-IGF-1 neutralizing antibodies did not show any effects (Fig. 6B and C).

## Discussion

Unlike most organs, the majority of mammary gland development occurs post-natally and subsequently completes during pregnancy and lactation. A mature breast has undergone tree-like ductal morphogenesis by epithelial cells that are surrounded by stromal cells and eventually fill the fat pad, which together form complex interaction networks. Normally, enlargement of the breasts during puberty and pregnancy is a physiological phenomenon. However, macromastia occurs when this process is too pronounced. Anne Dancey undertook a comprehensive literature review of the terms ‘gigantomastia, macromastia, breast hypertrophy’ and proposed the definition of gigantomastia as excessive breast growth of over 1500 g/breast, which can be classified into three subgroups: idiopathic, hormone stimulated and drug induced [1]. Dafydd proposed that gigantomastia should be defined as excess breast tissue over 3% of the patient's total bodyweight [2]. By either definition, the four patients involved in this study qualified for the diagnosis of gigantomastia or macromastia. All of these cases were spontaneous and quiescent at the time of presentation. In the absence of relentless growth during puberty or pregnancy and other obvious precipitating factors (*e.g*. excess weight, drug induced), we define these conditions as true macromastia. There has been limited investigation of this condition. Sun *et al*. [4] found a significant increase in oestrogen receptor expression in the mammary glands of breast hypertrophy patients compared with that in micromastia patients. Li *et al*. [18] detected genome-wide expression of miRNAs and mRNAs in macromastia patients and annotated their functions according to alterations in their expression. Their studies suggest that APK, Wnt and neurotrophin signalling pathways may play important roles in macromastia. However, insufficient attention was paid to the stroma. There are increasing data emphasizing the interactions between cell–cell or cells and their microenvironment in the proliferation, differentiation, polarity and even the invasive capacity of mammary epithelial cells [19–23]. Thus, we aimed to determine whether stromal cells are involved in epithelial actions in breast hypertrophy.

We used a classic method to isolate and culture primary mammary epithelial and stromal cells, and then developed a co-culture model in Matrigel to provide new insights into the mechanisms underlying mammary gland overgrowth. Our procedure for isolation of single cell suspensions from human mammary glands combined enzymatic digestion and Percoll gradient centrifugation [11,24]. After dissociation of the mammary gland, we harvested two major cell populations distributed at different levels in the discontinuous Percoll gradient according to their densities. The fraction in the interface of the two Percoll densities was a population of epithelial cells that was up to 95% positive for the epithelial-specific marker CK18. Another fraction composed of stromal cells was characterized by 95% positivity for vimentin. Morphological studies conducted on primary cell cultures from these two fractions further confirmed these findings. The procedure resulted in high yields and cell viability of monodispersed cell populations that could be subcultured for several passages.

Human mammary epithelial cells *in vivo* form a complex architectural arrangement that encompass cell–cell and cell–matrix interactions. Cell culture on plastic substrates does not support cell interactions and may fail to provide an accurate model for cellular behaviour *in vivo*. Efforts to remedy this shortcoming have been attempted in a number of studies using a variety of co-culture methods and extracellular matrix extracts to model the *in vivo* cellular microenvironment [19–21,25–27]. In the present study, we developed an *in vitro* model of mammary glands, which allowed the maintenance and differentiation of both epithelial- and stromal-derived primary cells within a defined 3D Matrigel. By recapitulating human pathological cells in the 3D milieu, we believe that the physiological conditions were accurately mimicked for better clarification of the interactions between stromal and epithelial cells in breast hypertrophy. This is the first report to use Matrigel primary co-culture conditions to study the pathogenesis of macromastia.

Compared with non-macromastic stromal cells, we found that alveolar morphogenesis of epithelial cells from either a macromastia or non-macromastia origin was enhanced in the presence of macromastic stromal cells. The final alveolar morphology could differ even in co-cultures with the same stromal cell population, according to the origin of the epithelial cells. These results may be related to the proliferation of epithelial cells, which increased in the presence of macromastic stromal cells. Consistent with the results of alveolar morphogenesis, the [^3^H] thymidine incorporation assay and Ki67 staining showed that CM from macromastia stromal cells stimulated the growth of epithelial cells from either macromastia or non-macromastia origin. Histologically, the breast tissue of macromastia mainly shows increased growth in the stromal tissue with pseudoangiomatous stromal hyperplasia, and may also show some degree of epithelial hyperplasia with absent or poorly formed lobules [1,28–30]. This appearance appears to be contradictory to our results. Touraine *et al*. [30] considered that these pathological features were caused by a delay in performing the breast biopsy, which does not reflect the initiation of breast hypertrophy. The proliferation potential of epithelial cells could not be excluded in macromastia. We analysed the rapidly growing stromal cells which inhibited branching of the epithelial cells and caused poor formation of lobules.

The effect of macromastic stromal cells on alveolar morphogenesis and epithelial cell proliferation may be caused by aberrant regulation of one or more growth factors. Secretion of HGF, which is known for oestrogen-induced proliferation and alveolar morphogenesis of epithelial cells in the breast [7,31–33], may be increased in macromastic stromal cells. Other growth factors produced in the mammary gland, which play an important role in the regulation of epithelial proliferation such as EGF and IGF-1 [34–36] could also be overexpressed by macromastic stromal cells. The results of immunoblotting for HGF, EGF and IGF-1 in CM from macromastic stromal cells showed significant increases of HGF and IGF-1 (5.4- and 1.8-fold, respectively) compared with that in CM from non-macromastia stromal cells, while the difference of EGF was not significant (1.12-fold). In antibody neutralization assays, we found that only an anti-HGF antibody attenuated the proliferative activity of CM from macromastic stromal cells, whereas anti-EGF and anti-IGF-1 neutralizing antibodies had no effect. Based on these results, abnormal regulation of HGF secretion in mammary stroma may be a critical factor in breast hypertrophy.

‘Stromal cells’ is a general term for several cell types, including fibroblasts, myofibroblasts, endotheliocytes, leucocytes, adipocytes and telocytes. Telocytes, also known as interstitial Cajal-like cells [37–44], were recently identified in the mammary stroma and characterized as possessing two or three moniliform cytoplasmic processes, which establish an ‘intercellular bridge’ in the microenvironment [45,46]. These cells are located among the tubule-alveolar structures and might play a pivotal role in the interactions of epithelial and stromal cells in mammary hypertrophy, which remains to be investigated.
